# Frontiers in chemistry grand challenge: open communication to the world

**DOI:** 10.3389/fchem.2014.00042

**Published:** 2014-07-01

**Authors:** Steven L. Suib

**Affiliations:** Department of Chemistry, University of ConnecticutStorrs, CT, USA

**Keywords:** chemical sciences, chemistry, open access, future challenges, open publication

The excitement of making a new compound, discovering new characterization methods, and applying these systems for practical applications is a feeling that many of us as chemists and scientists markedly enjoy. Yet how can we pass along these experiences to younger generations? One major way involves communication of our results in written forms that others can experience over long periods of time, and use to generate new and exciting ideas.

The pleasure and process of writing a research article is a complicated one. We need to learn to communicate so that a broad audience can understand what we are thinking, and most of us master this process by trial and error. The ability to have better feedback through, for example, the mechanism afforded by *Frontiers*, to clarify our thoughts and ideas, certainly should lead to improved writing and communication.

The subject of Chemistry is a very broad and exciting one. Chemistry evolved from alchemy and atomism to the chemical science that we know today, central to so many disciplines. There are clear examples of the current and future prospects of studying Chemistry ranging from studies of the nature and birth of the Universe to infinitesimal detail of how atoms, ions, and molecules come to ether at a very small scale. Chemistry is central to all research endeavors including the very current topics of energy, medicinal, pharmaceutical and biological chemistry, materials, and environmental research.

The world is and will be facing a variety of difficult problems that center around energy, resources, education, health, and climate. Chemists have the ability and knowledge to understand these problems, to design and carry out experiments to gain more fundamental insight, and to help design new strategies, materials and devices that will help humans survive and live better, longer, and happier.

An important first step in chemists helping our world is writing our results of experiments, writing about ideas to pursue for the future, and enhancing understanding of these complex areas of need. Authors should also not be reticent to publish disconfirming results or studies on the non-reproducibility of previously published work in journals that allow the general growth of knowledge and the exchange of information between research groups around the world.

Writing of research papers can readily lead to confusion if reviewers and editors are not conscientious. It is easy to spew out the everyday acronyms of instruments and methods that we use constantly without defining these. It is easy to mix results (facts) and discussion (opinions). It is easy to incorporate drawings, diagrams, and photos that are not of high quality. It is our duty as editors, reviewers, and authors to not take the easy route but rather to put in the effort to do things the right way. The right way is quite time consuming, but certainly well worth the effort. Open access journals, especially those like *Frontiers* that allow dialog between authors, reviewers, and editors while protecting anonymity of referees during the review process provide a unique environment for generation of articles that are more clear and useful due to such open conversation.

Publishers of Chemistry articles have very different ideas regarding methods and the structure of publications, and the concept of *Frontiers* of open access as well as outstanding feedback is currently unique. In the world of chemists, scientists, and engineers, this is akin to doing experiments. Open access and the novel pre-publication feedback implies open minds of such publishers. At the same time, clear and novel procedures regarding publishing of such articles, which are in their infancy as regards published articles is needed and likely will evolve in time.

The role of editors is that of watch keeper of the written record. Our duty of editing is to provide information, suggestions regarding protocols, to define areas of research interest, to enhance quality and visibility of articles and journals, and to do so in a timely manner. Editors need to take time to make sure reviews are fair, that enough and quality reviews are received, and that the papers to be accepted are complete, interesting, and unique.

The role of reviewers is also multi-fold. It is ideal that a reviewer is an expert in the field of expertise of the article being reviewed. However, that is not always the case since much of what we do as chemists is truly unique. A key need is a timely review. We all submit papers and we all appreciate receiving reviews in a reasonable time, especially at the current evolving rate of science and research. Reviewers need to provide details of ways to improve articles especially in the area of communication and understanding of what is trying to be written and disseminated by authors. Often what we write and what we intend to say are very different. Suggestions on improvements are quite different than simple criticism and it seems appropriate to start expecting collaboration between scientists even during the review process to publish great articles.

Finally and most importantly we come to authors. Without authors and submitted research articles there are no journals nor adequate transfer of information to future generations. Authors need to decide when they have enough clear results to tell a story. They need to spend the time to write as clearly as possible so that as many people from different walks of life can understand what they want to communicate. We are all continuing to learn how to write clearly and effectively. This is a difficult task because most of us have been well trained in skills of doing experiments but not in skills regarding excellent writing.

I look forward to working with all of the publishers, editors, reviewers, and authors of *Frontiers in Chemistry*. This is a very exciting and opportune time for chemists, scientists, and engineers who will make significant strides to solve the problems of our world. Our Grand Challenge in *Frontiers in Chemistry* is to promote the publication of exciting, clear, and well-reviewed manuscripts of the highest quality and relevance to the area of chemistry and in particular to help solve current and future fundamental and applied problems that we all face.

## Conflict of interest statement

The author declares that the research was conducted in the absence of any commercial or financial relationships that could be construed as a potential conflict of interest.

